# MRI compatibility of orthodontic brackets and wires: systematic review article

**DOI:** 10.1186/s12903-022-02317-9

**Published:** 2022-07-19

**Authors:** Adrienn Dobai, Fanni Dembrovszky, Tamás Vízkelety, Péter Barsi, Fanni Juhász, Csaba Dobó-Nagy

**Affiliations:** 1 Department of Oral Diagnostics, Faculty of Dentistry, Semmelweis University, Szentkirályi u. 47, Budapest, 1088 Hungary; 2 Institute for Translational Medicine, Medical School, University of Pécs, Szigeti Street 12, Pecs, 7024 Hungary; 3 Dento-Cura Private Practice, Kálvin Street 3, Budapest, 1053 Hungary; 4 Department of Neuroradiology, Medical Imaging Centre, Semmelweis University, Balassa Street 6, Budapest, 1083 Hungary; 5 Department of Paediatric Dentistry and Orthodontics, Faculty of Dentistry, Semmelweis University, Szentkirályi u. 47, Budapest, 1088 Hungary

**Keywords:** Orthodontics, Fixed orthodontics appliances, Orthodontic bracket, Orthodontic wire, Orthodontic retainers, Magnetic resonance imaging

## Abstract

**Background:**

Before the magnetic resonance imaging (MRI) examination fixed orthodontic devices, such as brackets and wires, cause challenges not only for the orthodontist but also for the radiologist. Essentially, the MRI-safe scan of the fixed orthodontic tools requires a proper guideline in clinical practice. Therefore, this systematic review aimed to examine all aspects of MRI-safe scan, including artifact, thermal, and debonding effects, to identify any existing gaps in knowledge in this regard and develop an evidence-based protocol.

**Methods:**

The Preferred Reporting Items for Systematic Reviews and Meta-analysis (PRISMA) statement was used in this study. The clinical question in “PIO” format was: “Does MRI examination influence the temperature of the orthodontic devices, the size of artifacts, and the debonding force in patients who have fixed orthodontic bracket and/or wire?” The search process was carried out in PubMed, PubMed Central, Scopus, and Google Scholar databases. The search resulted in 1310 articles. After selection according to the eligibility criteria, 18 studies were analyzed by two reviewers. The risk of bias was determined using the Quality In Prognosis Studies tool.

**Results:**

Out of the eligible 18 studies, 10 articles examined the heating effect, 6 were about the debonding effect, and 11 measured the size of artifact regarding brackets and wires. Considering the quality assessment, the overall levels of evidence were high and medium.

The published studies showed that heating and debonding effects during MRI exposure were not hazardous for patients. As some wires revealed higher temperature changes, it is suggested to remove the wire or insert a spacer between the appliances and the oral mucosa. Based on the material, ceramic and plastic brackets caused no relevant artifact and were MRI-safe. Stainless steel brackets and wires resulted in susceptibility artifacts in the orofacial region and could cause distortion in the frontal lobe, orbits, and pituitary gland. The retainer wires showed no relevant artifact.

**Conclusions:**

In conclusion, the thermal and debonding effects of the fixed orthodontic brackets and wires were irrelevant or resoluble; however, the size of the artifacts was clinically relevant and determined most significantly the feasibility of fixed brackets and wires in MRI examination.

## Introduction

Nowadays orthodontic treatments are commonly used not only in childhood but also in adulthood. The number of adult orthodontic cases has increased rapidly in the last few years. The results of a study by the American Association of Orthodontists demonstrated that the number of adults undergoing orthodontic treatment in the US and Canada increased by 16% from 2012 to 2014 [1]. In parallel with this trend, the number of magnetic resonance imaging (MRI) examinations in the world has also risen [2]. These two trends can be relevant as many times patients with orthodontic apparatus need MRI examinations. Some of the orthodontic appliances are removable; therefore, it is possible and necessary to remove them before the MRI examination; however, the fixed orthodontic devices often cause a dilemma.

The fixed appliances consist of multibracket appliances (e.g., brackets, bands, archwires, ligature wires), retainers, expanders (e.g., hyrax and palatal and lingual arches), screws (e.g., bone screws and orthodontic mini-implants). The most frequently used tools are brackets and wires, which are connected to each other. The possibility of undergoing MRI examinations while having orthodontic brackets and wires has remained an unresolved issue. In the literature, there is only one guideline, which is complex, for using fixed orthodontic apparatus in MRI developed by an official medical society [3]. To date, in the practice in most of the cases radiologists ask to remove every orthodontic tool before the MRI scan even though some orthodontic devices could be scanned safely. Another problem is that the materials of the fixed orthodontic appliances are often unknown, making them removed [4]. However, this process is not easily accomplishable and takes considerable time. Furthermore, the removal procedures of bonded devices can potentially damage the enamel and are time-consuming, uncomfortable for the patient, and costly [5]. Due to these reasons, orthodontists and radiologists are challenged to decide whether to keep or remove orthodontic appliances prior to an MRI examination.

Essentially, an MRI-safe scan of fixed orthodontic tools requires proper clinical practice guidelines. Therefore, this systematic review aimed to examine all aspects of MRI-safe scan, including artifact, thermal, and debonding effects, to identify any existing gaps in knowledge in this regard and develop an evidence-based protocol.

## Methods

### Protocol and registration

This systematic review was conducted in accordance with the Cochrane Collaboration [6] and it followed the Preferred Reporting Items for Systematic Reviews and Meta-Analyses statement [7]. The study protocol was not registered. The clinical question in “PIO” format (Table 1) in our study was: “Does MRI examination influence the temperature of the orthodontic devices, the size of the artifacts, and the debonding force in patients who have fixed orthodontic brackets and/or wires?”.

**Table 1:** Search question using PIO model

PIO	
Problem	Fixed orthodontic wire or bracket
Intervention	MRI examination
Outcome	Increased temperature and artifacts and irrelevant debonding effect

### Search method for identification of studies

For the identification of the studies included in this review, we devised the search strategy for each database. The search strategy consisted of using a combination of controlled vocabulary and free text terms. A detailed search was carried out on PubMed, PubMed Central, Scopus, and Google Scholar databases using the following keywords: “Magnetic resonance imaging” (MRI), “Orthodontic bracket”, “Orthodontic wire”, “Bracket”, “Orthodontic application”, and “Orthodontic device”. The search period was from 1970 to 2021, and the search process started on 28.07.2021.

### Inclusion and exclusion criteria

**Fig. 1:**
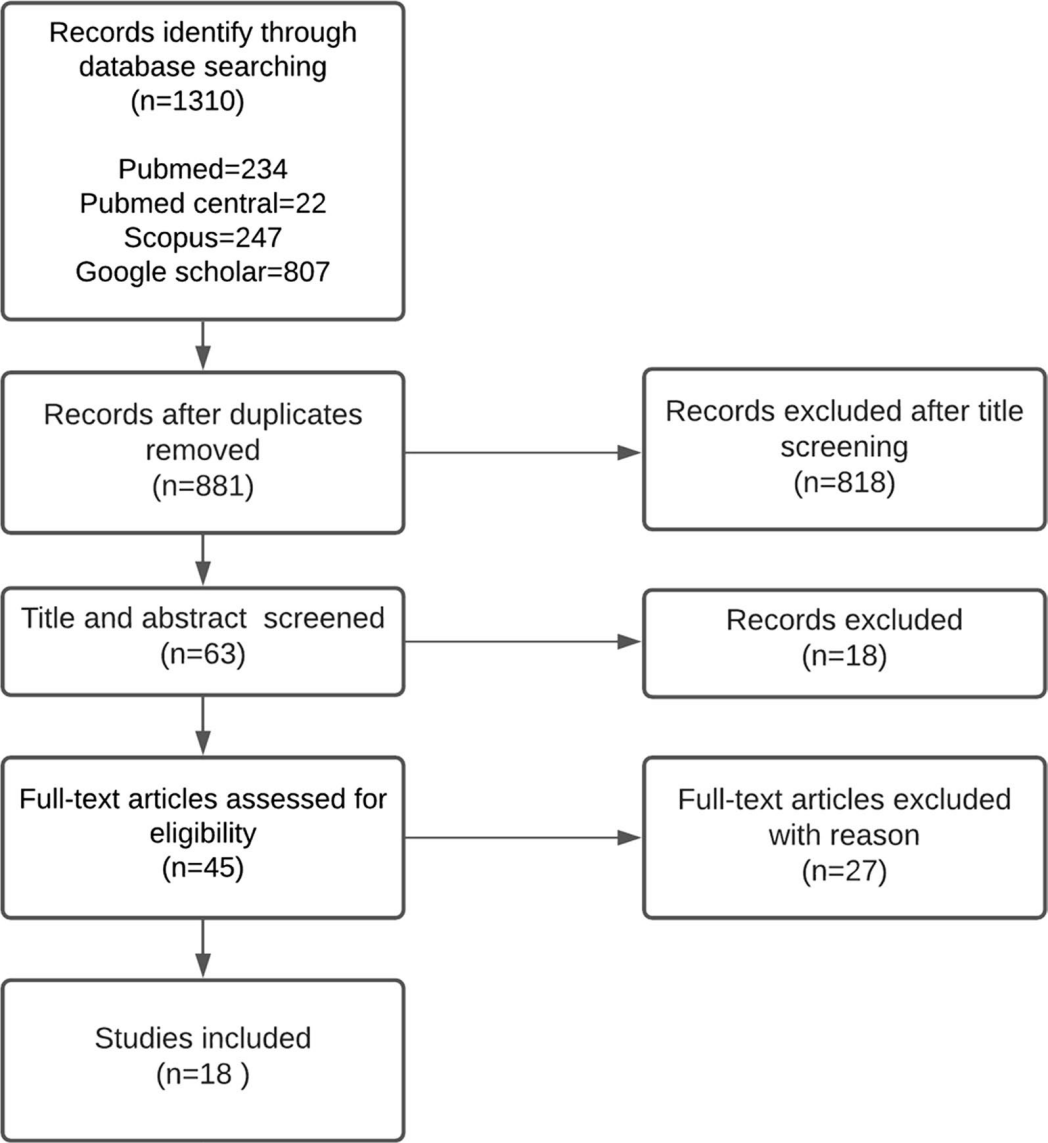
Flowchart outlining the protocol adopted in this systematic review based on the Preferred Reporting Items for Systematic Reviews and Meta-analyses (PRISMA)

Studies with different designs (e.g., clinical trials, cohort studies, case–control studies, cross-sectional studies, and prospective and retrospective studies) reporting the relationship of orthodontic bracket or wires with MRI regarding image quality, thermal effect, and debonding effect were included in this study. However, reviews, editorials, letters, case reports, and historical articles as well as research using 0.5 T MRI were not included. The excluded studies also consisted of the articles published in languages other than English and those whose only the abstracts were written in English and lacked enough information about the materials of the examined brackets or wires. Moreover, by evaluating the artifact, only in vivo studies were selected as the radii of the artifact measured in in vitro researches using different study casts are hardly adaptable in the clinical practice (Fig. 1).

### Screening process and data collection

A total of 1310 papers were identified through the systematic search. After excluding duplicate studies, 881 papers remained. Preliminary selected papers were reviewed according to the inclusion/exclusion criteria, and irrelevant reports were identified by their title/abstract and excluded by the first reviewer (AD). As a result, 836 irrelevant papers were excluded. 59 of 836 articles were published only in languages other than English (most of them were written in Chinese). Moreover, there were five review articles, three case reports/ survey and one author response, which were excluded because of the type of the article, although, the topics were proper. The other excluded papers did not focus on the examined questions. For the next stage, the full texts of 45 studies were screened, and the papers were independently examined in duplicate by two reviewers (AD and TV) to confirm eligibility. Finally, a total of 18 papers were identified by the two reviewers as eligible for this review (Fig. 1).

### Quality assessment (risk of bias)

The methodologic quality of included studies was assessed independently and in duplicate by two reviewers (AD, TV) using the Quality In Prognosis Studies (QUIPS) tool [8]. Each domain in the tool was allocated to one of the following judgments: low, moderate, or high risk of bias or not applicable.

## Results

### Thermal effect

In the literature, some studies determined a minimal increase in the temperature (i.e., < 1 °C) of stainless steel (SS) and nickel-titanium (NiTi) brackets and wires during 3 T as well as 1.5 T MRI exposures [9, 10]. Hasegawa et al. used fixed brackets and wires to determine the temperature elevation at 3 T MRI. Based on the results of the mentioned study, although the temperature of orthodontic objects increased at the range of 1.56–2.61 °C, the researchers declared that a fixed orthodontic apparatus did not pose a risk to the patients; nonetheless, it was recommended to remove the wire or put a spacer between the appliances and the oral mucosa [9]. In another study, the effect of brackets and wires during 3 T MRI exposure was analyzed, and temperature changes were reported within acceptable ranges; however, the maximum difference was 3.2 °C in the groups of NiTi archwires and continuous SS ligature wires [11].

In 2019, an outstanding article was published, examining 220 orthodontic brackets with different wires using 3 T and 1.5 T MRI machines. Their results showed that the temperature changes were in the range of 0.42–1.74 °C for wires and 0.05–2.4 °C for brackets. Although these changes were statistically significant, they were clinically not relevant. Therefore, the researchers of the mentioned study concluded that these fixed orthodontic appliances presented a low risk with no difference between the 1.5 T and 3 T groups [12]. We found only one article in the literature which used a 7 T MRI machine for testing the heating effect of retainer wire, the results of which were indicative of only an irrelevant 1.5 °C increase during the study [13].

To sum up, the maximum temperature elevation was estimated at ≤ 1 °C in 42% of the selected articles, and in all studies the examined radiofrequency-induced heating near fixed orthodontic appliances demonstrated clinically insignificant temperature rising (i.e., < 5.6 °C) during the MRI examination (Table 2).

**Table 2:** Articles about radiofrequency heating effect in case of fixed orthodontic appliances

Title	Authors	Year	Examined objects	MRI	Conclusion/results
Evaluation of magnetic resonance imaging issues of titanium and stainless steel brackets	Linetskiy et al	2019	Titanium and stainless steal bracket	1.5 T	Maximal temperature elevation was 0.1 °C
Magnetic resonance imaging and its effects on metallic brackets and wires: Does it alter the temperature and bonding efficacy of orthodontic devices?	Sfondrini et al	2019	220 orthodontic brackets and wires: SS brackets, NiTi brackets SS wires, NiTi wires	1.5 T and 3 T	Temperature elevation was between 0.05 and 2.4 °C for brackets, 0.42–1.74 °C for wires No significant difference between 3 T and 1.5 T
Radiofrequency-induced heating near fixed orthodontic appliances in high field MRI systems at 3.0 Tesla	Regier et al	2009	SS brackets, SS palatal expander, lip bumper	3 T	Maximum temperature elevation was 0.2 °C. Negligible temperature elevation
Radiofrequency heating of metallic dental devices during 3.0 T MRI	Hasegawa et al	2013	Bridges, fixed prosthesis, metal bracket + wire + molar band	3 T	Temperature elevation was between 1.56 and 2.61 °C by orthodontic appliances
Effect of orthodontic brackets and different wires on radiofrequency heating and magnetic field interactions during 3-T MRI	Görgülü et al	2014	SS brackets, NiTi brackets SS archwires, NiTi archwires SS ligature wire	3 T	Maximal temperature elevation was between 1.8 and 3.2 °C
Assessing the MR compatibility of dental retainer wires at 7 Tesla	Wezel et al	2014	Retainer wire	7 T	Maximal temperature elevation was 1.5 °C

### Debonding effects

In the literature, there were only eight articles about the debonding effect of magnetic fields on orthodontic appliances. This effect can be examined by the shear bond strength test and deflection angle test.

Klocke et al. examined 32 orthodontic wires in two studies using 1.5 T and 3 T MRI, respectively. The findings of the aforementioned study revealed that the archwires made of NiTi, titanium-molybdenum, and cobalt-chromium and different ligature wires showed no or negligible forces in the magnetic field. In almost all archwires and retainer wires made of steel, the deflection angle was around 90° at both magnetic fields. In the steel and brass ligature wires, the deflection angles were lower than 4° (1.5 T) or 14.67° (3 T). The comparison of the results of these two studies shows that the deflection angles were very similar by 3 T MRI, compared to the 1.5 T MRI [14, 15].

In 2019, Sfondrini examined 220 orthodontic appliances (brackets and wires) and demonstrated that there was no relevant debonding effect tested with the shear bond strength test and there were no significant differences between the different materials and sizes of wires [12].

Görgülü et al. also showed the minimum deflection angle for the metal brackets (13°) and higher values for NiTi and SS wires (62° and 71°, respectively) [11]. In another study, Wezel et al. examined the debonding effect of a 7 T machine on the retainer wires, in which the deflection angles were found at the range of 0°–56° [13] (Table 3).

**Table 3:** Articles about debonding effect in case of fixed orthodontic appliances

Title	Authors	Year	Examined objects	MRI	Conclusion/results
Magnetic resonance imaging and its effects on metallic brackets and wires: Does it alter the temperature and bonding efficacy of orthodontic devices?	Sfondrini et al	2019	220 orthodontic brackets and wires: SS brackets, NiTi brackets SS wires, NiTi wires	1.5 T and 3 T	Shear bond strength values were between 12.04 and 35.43 MPa
Magnetic Field Interactions of Orthodontic Wires during Magnetic Resonance Imaging (MRI) at 1.5 Tesla	Klocke et al	2005	9 NiTi archwires 8 SS archwires 2 Co–Cr archwires 2 Ti–Mo archwires 7 SS ligature wires 1 brass ligature wires 3 SS retainer wires	1.5 T	Archwires from NiTi, Ti–Mo, Co–Cr and different ligature wires showed no or negligible forces in the magnetic field By archwires and retainer wire made of steel in almost all cases the deflection angle was 90°
Evaluation of magnetic resonance imaging issues of titanium and stainless steel brackets	Linetskiy et al	2019	Ti and SS bracket	1.5 T 4.7 T	Deflectaion angle was 0° by the Ti bracket and nore than 89° by the SS bracket
Magnetic forces on orthodontic wires in high field magnetic resonance imaging (MRI) at 3 T	Klocke et al	2006	9 NiTi archwires 8 SS archwires 2 Co–Cr archwires 2 Ti–Mo archwires 7 SS ligature wires 1 brass ligature wires 3 SS retainer wires	3 T	Archwires from NiTi, Ti–Mo, Co–Cr and different ligature wires showed no or negligible forces in the magnetic field By archwires and retainer wire made of steel in almost all cases the deflection angle was 90°
Effect of orthodontic brackets and different wires on radiofrequency heating and magnetic field interactions during 3-T MRI	Görgülü et al	2014	SS brackets, NiTi brackets SS archwires, NiTi archwires SS ligature wire	3 T	Deflection angle (mean) for the brackets: 13° Deflection angle (mean) for NiTi wires 62° Deflection angle (mean) for stainless steel wire 71°
Assessing the MR compatibility of dental retainer wires at 7 Tesla	Wezel et al	2014	12 retainer wires	7 T	Deflection angles for retainer wires were between 0° and 56°

### Artifacts

Some factors influence the degree of artefacts during the MRI examination of patients with a ferromagnetic material, including the strength of the magnetic fields, the degree of ferromagnetism, and the geometry and location of the material [16]. Regarding the strength of the magnetic fields, 3 T MRI has a higher signal-to-noise ratio, which is well accepted for such applications as high‐resolution brain imaging; however, 3 T magnetic field may have some drawbacks, including increased levels of imaging artifacts. Although, every imaging artifact observed at 3 T can also be present at 1.5 T, artifacts are more prominent at 3 T [17], which can influence the feasibility of orthodontic appliances.

A common fact is that orthodontic appliances can cause artifacts not only in the facial region [18] but also in the brain and spine [19]. The results of some studies have revealed that magnetic permeability is a good predictor of artifact size [20, 21].

Elison et al. studied artifacts caused by brackets made of four different materials in different cranial regions. Accordingly, ceramic, titanium, and plastic apparatuses showed acceptable, minimal distortion; nevertheless, in the case of SS, the artifact was relevant and significant in the following regions: base of the tongue, the body of the mandible, hard palate, orbits, nasopharynx, pituitary gland, frontal lobe, and temporal lobe. It was also reported that the mean distortion score in the facial regions was high, while in the frontal and temporal lobes and pituitary gland it was almost acceptable [22]. Asano et al. found that the SS bracket caused relevant artefacts in the frontal, occipital, and temporal lobes, ventricle, brain stem, pituitary gland, and cerebellum in the brain MRI examinations [23]. Sonesson et al., who compared the image quality between 1.5 and 3 T MRI machines, emphasized that in 3 T MR images, the pituitary gland, nasopharynx, and orbit were more distorted [24]. The findings of some studies have indicated that SS elements should be removed before the MRI examination of the oral and facial regions [25, 26]. Cassetta et al. emphasized that during brain MRI, there were artifacts in the frontal lobe, at the cervical segments of the spinal cord, and in the bone marrow on the T2 images, which could mimic pathology. Regarding the temporomandibular joint (TMJ), they found diagnostically proper images [25]. Costa et al. examined brain MRI using epilepsy protocol and reported that the metallic braces caused artefacts, especially in the frontal and temporal lobes by every axis [19]. Beau et al. focused only on brackets as the wires could easily be removed prior to MRI. In this study, the titanium brackets, ceramic brackets with metal slots, and SS retainers caused relevant artifacts only in the oral region. Still, in contrast with the previous study, the SS brackets caused distortion also in the TMJ and posterior cranial fossa [27].

According to the findings of a study by Wylezinska et al., SS degraded image quality beyond clinical acceptability not only in the oral cavity but also in the TMJ and pituitary gland; therefore, it should be removed prior to imaging [28]. Okano et al. investigated only TMJ diagnosis with and without orthodontic appliances. They found a decline in diagnostic accuracy as the amount of metal increased. In this regard, the accuracy values with a metal bracket having and lacking SS wires were obtained at 60% and 70%, respectively. Consequently, they suggested the use of ceramic brackets in the front teeth and direct bonding tubes in the molar teeth and the removal of archwires [29] (Table 4).

**Table 4:** Articles about artifacts in case of fixed orthodontic appliances

Title	Authors	Year	Examined objects	MRI	Uncertain anatomical region
Influence of common orthodontic appliances on the diagnostic quality of cranial magnetic resonance images	Elison et al	2008	SS bracket Ceramic bracket Plastic bracket Titanium bracket	1.5 T	SS brackets: base of the tongue, body of mandible, hard palate, orbits, nasopharynx, pituitary gland, frontal lobe, temporal lobe
Magnetic resonance imaging artifact and fixed orthodontic attachments	Beau et al	2017	SS bracket Ceramic bracket with metal slot Titanium bracket SS retainer	1.5 T	TI, Ceramic brackets, SS retainer: oral cavity SS bracket: oral cavity, maxillary sinus, posterior cerebral fossa, TMJ
Magnetic resonance imaging diagnosis of the temporomandibular joint with orthodontic appliances	Okano et al	2003	Ceramic bracket, Metal bracket, SS wires	0.5 T	SS wires: TMJ
Impact of orthodontic appliances on the quality of craniofacial anatomical magnetic resonance imaging and real-time speech imaging	Wylezinska et al	2015	Ceramic brackets, Ceramic brackets with metal slot, SS brackets, SS archwires SS molar band	1.5 T	SS bracket, molar band: oral cavity, pituitary gland, TMJ
Magnetic resonance imaging artifacts caused by brackets of various materials-An in vivo study	Razdan et al	2016	Ceramic brackets, Composite brackets SS brackets	1.5 T	SS brackets: Entire midface and lover facial region except of posterior wall of pharynx and orbit
Influence of metal artifact by orthodontic appliances on brain MRI	Asano et al	2016	SS bracket, Resin bracket, ceramic bracket, Ti bracket	1.5 T	Examined only brain SS bracket: frontal, occipital and temporal lobe, ventricule, brain sterm, pituitary gland, cerebellum
Orthodontic appliances and MR image artefacts: An exploratory in vitro and in vivo study using 1.5-T and 3-T scanners	Sonesson et al	2021	SS bracket, Ni free bracket, Ti bracket, Herbst appliances, fixed retainer, Expander	1.5 T and 3 T	SS, Ni free: maxilla, mandible, tongue on 1.5 T in addition pituitary gland, eye globe, nasopharynx on 3 T Ti bracket: only minor artefact by maxilla and mandible
Whole-Brain Functional and Diffusion Tensor MRI in Human Participants with Metallic Orthodontic Braces	Miao et al	2020	SS bracket with Ti archwire	3 T	SS bracket by functional MRI: Orbitofrontal and ventromedial prefrontal cortex using SS bracket by DTI MRI: Inferior frontal lobe
The effects of a common stainless-steel orthodontic bracket on the diagnostic quality of cranial and cervical 3 T- MR images: a prospective, case–control study	Cassetta et al	2017	20 SS brackets without archwires, 20 SS brackets with NiTi wires, 20 SS brackets with SS wires	3 T	SS brackets: paranasal, cervical, head and neck
Assessing the MR compatibility of dental retainer wires at 7 Tesla	Wezel et al	2014	12 retainer wires	7 T	Retainer wires: Tongue
Artefacts in brain magnetic resonance imaging due to metallic dentel object	Costa et al	2009	Dental implant Orthodontic braces	not identified	Mettalic braces: (only brain was examined) Artefacts were by every axises

### Quality assessment (risk of bias)

**Fig. 2:**
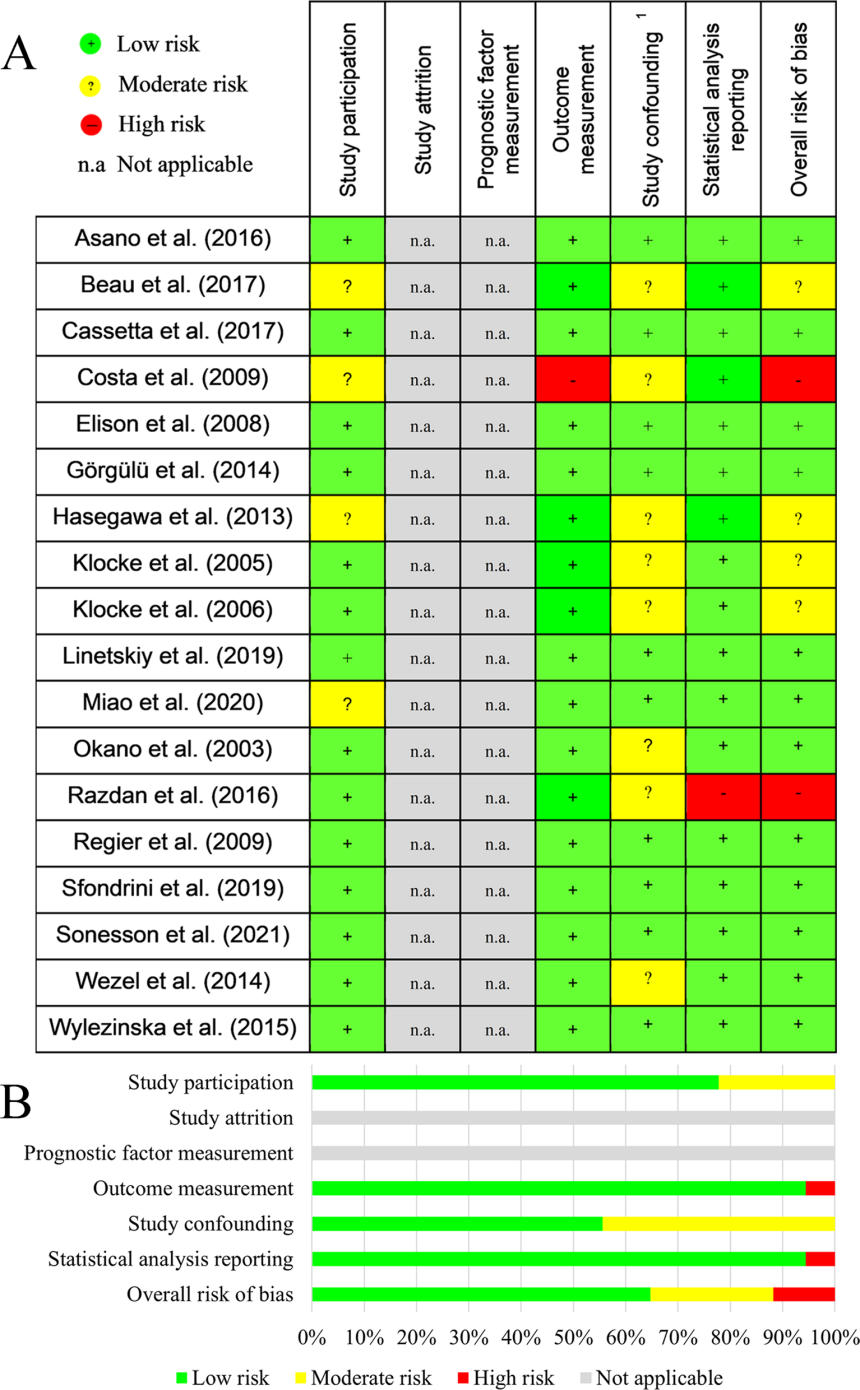
Risk of bias assessment on study level (**A**) and across studies (**B**). 1: Assessed confounding factors are dental materials, MRI sequences, magnetic force

The QUIPS tool was used to assess the risk of bias. Of the six bias domains, only five (i.e., Study participation, Outcome measurement, Study confounding, Statistical analysis and reporting, and Overall risk of bias) were applicable in this review. Figure 2 depicts the ratings of each article. The study participation category showed low risk in 14 cases and moderate risk in 4 cases. Of the 18 analyzed studies, 1 received high risk in the category “statistical analysis” and 1 had high risk in the “outcome measurement” category. By the analysis of the study cofounding, 44% and 66% of the articles showed moderate and low risks of bias, respectively (Fig. 2).

## Discussion

Although in dentistry there are official guidelines about X-ray and cone-beam computed tomography indications, such as SEDENTEXCT [30], currently there is no consensus about MRI guidelines. In the literature, we found two review articles in relation to this topic. In one of these studies, Chockattu et al. examined the interaction between dental materials and MRI without focusing on orthodontic devices [31], while in another study, Hasanin et al. reviewed the effect of orthodontic appliances on head and neck MRI; however, they analyzed only the change of image quality [32]. As the results of some studies have demonstrated that metal artifacts on computed tomography (CT) are more troublesome than on MRI images, the MRI can be favorable for head and neck diagnostics [33]. Consequently, creating a protocol that simplifies the referral order to MRI examinations in the case of patients with orthodontic appliances would be practical.

It is accepted as a rule that all removable orthodontic apparatus should be removed before MRI examination. In the case of fixed orthodontic devices, it is difficult to create a standard rule since the material causes artifact, thermal, and debonding effects, and the region of interest determines the safe scan of orthodontic apparatus by MRI.

The temperature changes in the oral environment and dental tissues cannot be directly transmitted to the pulp as enamel and dentin structures cover it, and recently the CENELEC standard (prEN45502-2–3) specifies the vitality boundary of the pulp, which is 5.6 °C. To summarize these results, we can conclude that the heating of fixed orthodontic brackets and wires during 3 T or 1.5 T MRI exposure is not hazardous for patients. Considering that some wires showed higher temperature changes and these objects are easily removable, the removal of the wire or the insertion of a spacer between the appliances and the oral mucosa is suggested. According to these articles, there are no significant differences in the degree of heating between the 1.5 T and 3 T MRI machines.

The literature was consistent on the debonding effect of the fixed orthodontic appliances. According to the ASTM international 2052-02 standard [34], any steel tool with a deflection angle of ≥ 45° should be classified as MRI-unsafe. Considering this, the appliances from ceramic, plastic, NiTi, titanium-molybdenum, and cobalt-chromium do not present a risk. Stainless steel brackets have variable translational and rotational forces; nevertheless, these forces are lower than the forces generally necessary for dislodging these bonded orthodontic brackets from tooth surfaces. In the case of SS archwires, the debonding forces can be over the standard limit [11, 14, 15]. As those appliances can be easily removed, their removal is recommended before an MRI examination. In contrast, the ligature wires do not pose the risk of debonding due to their minimal size.

Considering the artifacts, a general fact is that a more significant distance between the orthodontic apparatus and the interested region would lead to fewer artifacts [22]. Based on the material, ceramic and plastic brackets cause no relevant artifacts and are MRI safe [35]. Stainless steel brackets caused magnetic susceptibility artifacts in the oral and facial regions and can also cause distortion in the frontal lobe [22, 25, 27]. In head and neck imaging, for example the MRI of the paranasal sinuses, it is necessary to remove the SS brackets and wires because they cause a relevant distortion and a possible debonding effect; however, titanium brackets can be acceptable. The oral cavity is one of the most problematic regions for CT and MRI examinations as well. In the cases of the diagnostics of tongue cancer or other oral disorders, we can use only ceramic or plastic brackets as titanium also causes relevant artefacts in the oral region [27, 28, 36].

For the MRI examination of the TMJ, the use of SS brackets is questionable [25, 28, 29]. The presence of SS wires increases the risk of image distortion; therefore, the SS wire needs to be removed, and titanium wires are also suggested to be eliminated. Stainless steel wires and brackets have a risk, although low, of distorting the images of the TMJ region. According to the literature, the final recommendation is to always remove SS wires and if possible, SS brackets are also suggested to be removed.

During brain MRI, the accurate diagnostics of the frontal lobe, orbits, and pituitary gland in cases of demyelinating disease, stroke, or pituitary adenoma requires the removal of the SS brackets. In those patients, who presumably need periodic MRI examinations of the head, brain, or cervical spine during the orthodontic treatment, the use of ceramic, plastic, or titan brackets is suggested.

As the SS archwires are easily removable, numerous studies suggest removing them [9]. Nevertheless, the authors agree that all retainer wires can be left in the mouth as long as the target region does not expressly involve the tissues of the oral cavity [13, 27].

**Fig. 3:**
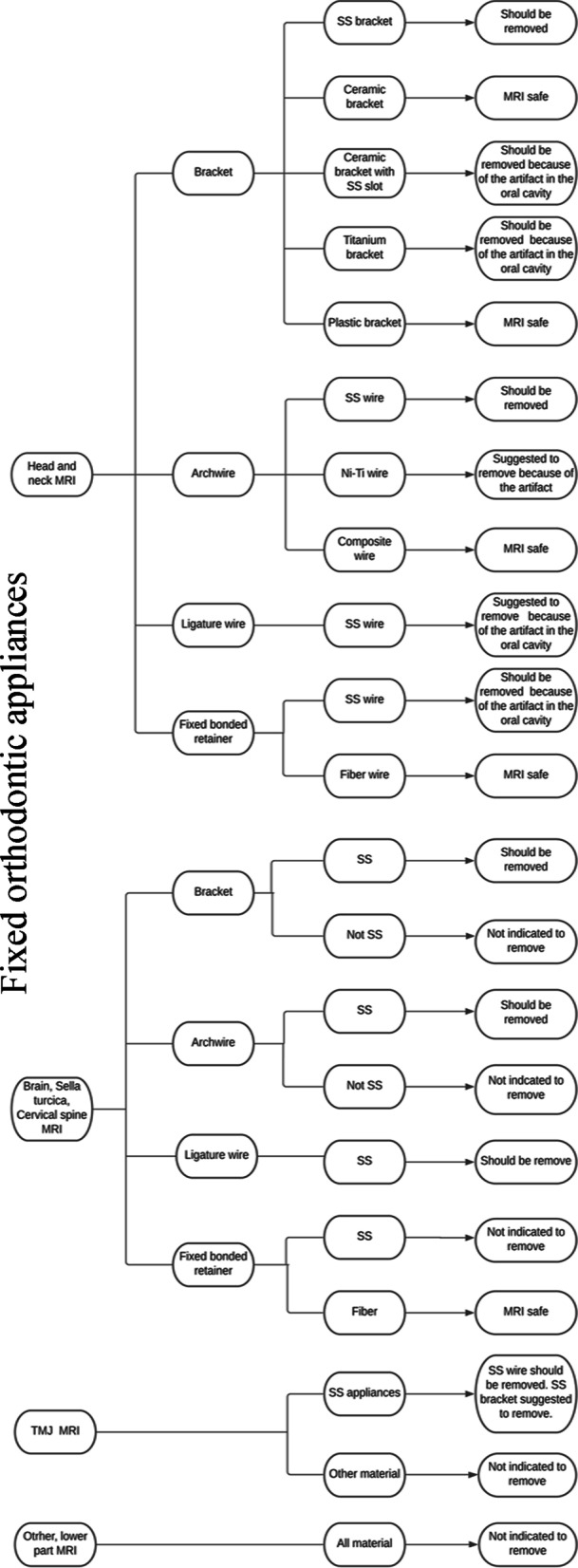
Suggested guideline for the various orthodontic appliances based on material and the region of the MRI

Figure 3 illustrates the summary of the review. Some general considerations can help reduce artifacts in fixed orthodontic apparatus and facilitate examinations:

1. It is preferred to use 1.5 T MRI in scanning the orofacial region because a higher field strength means higher sensitivity to motion artifacts [37].
2. It is recommended to use sequences less sensitive to susceptibility. Spin echo sequences are a good choice to reduce the artifacts [19, 38], and T1 weighted images are less susceptible than T2 weighted images. The gradient echo and echo planar imaging sequences are the most sensitive sequences [35, 38].
3. In all cases, the orthodontist needs to inform all patients in written form about the materials of the orthodontic appliances, whether the orthodontic appliances are MRI-safe, and the mandatory actions prior to MRI examination.

## Limitations of the review

As with any systematic review, there was the potential for selection bias. Although we used a comprehensive search strategy, it included only databases without the manual search. Only a small proportion of studies were published in other languages than English.

The heterogeneity among studies, particularly with respect to debonding effect, was a challenge as many studies presented different methods for the evaluation of debonding effects.

## Conclusion

The overall levels of evidence were high and medium. In conclusion, the thermal and debonding effects of fixed orthodontic brackets and wires were irrelevant or resoluble; however, the size of the artifacts was clinically relevant and it determined most significantly the feasibility of fixed brackets and wires in MRI examination.

## Data Availability

The datasets used and/or analyzed during the current study are available from the corresponding author on reasonable request.

## Abbreviations

MRI: Magnetic resonance imaging
NiTi: Nickel-titanium
PRISMA: Preferred Reporting Items for Systematic Reviews and Meta-analysis
QUIPS: Quality in prognosis studies
SS: Stainless steel
TMJ: Temporomandibular joint
